# Rapid detection of PAH gene mutations in Chinese people

**DOI:** 10.1186/s12881-019-0860-5

**Published:** 2019-08-05

**Authors:** Xin Zhang, Huan-Xin Chen, Chuan Li, Gui Zhang, Sheng-Yun Liao, Zhuo-chun Peng, Xiao-Ping Lai, Ling-Li Wang

**Affiliations:** 1 Mathematical Engineering Academy Of Chinese Medicine, Guangzhou University of Chinese Medicine, no. 232, Waihuandong Road, Guangzhou Higher Education Mega Center, Guangzhou, 510006 People’s Republic of China; 2 Shenzhen Yilifang Biotech Co. Ltd, A high-tech incsubator in Shenzhen hi-tech Zone, 2-301, Shenzhen, People’s Republic of China; 3 Cancercentrum Karolinska, SE-17176 Solna, Stockholm Sweden

**Keywords:** Phenylketonuria, Gene mutation, Single-tube multiplex PCR reaction, Reverse dot blot

## Abstract

**Background:**

Phenylketonuria (PKU) is an autosomal recessive genetic disease, caused by the phenylalanine hydroxylase (PAH) deficiency in the metabolic pathway, which prevents phenylalanine from being converted into tyrosine, leading to a large amount of phenylalanine discharged from the urine. Therefore, it is necessary to establish a simple, fast, accurate and reliable PKU molecular diagnostic method for clinical diagnosis.

**Methods:**

We established a novel diagnostic method by combining a single-tube multiplex PCR technique with molecular hybridization technique. The method was verified by DNA sequencing technology. The established new technology successfully detected 9 common PAH gene mutations in the Chinese population.

**Results:**

Double-blind analysis indicated that the diagnostic accuracy and specificity of the PKU sample were all 100%. Frequencies of single mutation R111X, R176X, Ex6–96A, R241C, R243Q, R252Q, Y356X, V399 V and R413P genotypes were 8, 0.5, 16.5, 1.5, 27, 4.5, 13, 10.5, 8.5% respectively.

**Conclusions:**

The established method of combing single-tube multiplex PCR with molecular hybridization technology can accurately and rapidly detect PAH gene mutations in Chinese and is suitable for screening of large PKU populations with clinical samples.

## Background

Phenylalanine hydroxylase (PAH) is an enzyme which is necessary to metabolize the amino phenylalanine to the amino acid tyrosine. It is a key enzyme in the phenylalanine metabolic pathway [1]. Phenylketonuria (PKU) is an autosomal recessive genetic disorder in which phenylalanine hydroxylase deficiency results in phenylalanine metabolic disorders [2]. If the child is not treated promptly after birth, it will cause severe retardation of intellectual development due to the hyper-toxic effects of hyperphenylalaninemia and intermediate metabolites in the central nervous system.

The *PAH* gene is located on chromosome 12q22-q24.1 and contains 13 exons and 12 introns encoding with a protein containing 452 amino acids. At present, more than 600 mutations of *PAH* have been reported worldwide, and more than 70 mutations have been detected in China, mostly on exon 6th, 7th, 11th, and 12th exon. Among them, the 9 mutations R111X, R176X, Ex6–96A > G, R241C, R243Q, R252Q, Y356X, V399 V, R413P are the most common types of PAH mutations in Chinese population [3–6]. The frequency and type of the *PAH* gene in different ethnicities and different regions are quite different and show great genetic heterogeneity, therefore, rapid detection of race-specific and common mutations is the focus of research. As a mature technology, reverse dot blot (RDB) technology has been successfully applied to the genotypic diseases such as thalassemia and Glucose-6-phosphate dehydrogenase(G6PD) deficiency [7, 8]. In this study, a molecular diagnostic technique that combined single-tube multiplex PCR and reverse-point hybridization were successfully established, for the simultaneous detection of 9 mutations in the PAH gene commonly found in Chinese populations.

## Methods

### DNA standard sample

A clinically tested blood sample was drawn and anticoagulated using EDTA-2Na-(Solarbio, China, Batch number: E8030). The DNA was extracted using the Qiagen Whole Blood Genomic DNA Extraction Kit (Qiagen, China, Batch number: 20150114). Thirty DNA standard samples of 30 PAH genotypes identified by DNA sequencing were 27 in 9 mutants and 3 in wild-type. The R243Q (G → A) genotype was an artificial plasmid used for the establishment of this method. (This study was submitted to and approved by Guangzhou University of Chinese medicine vhospital institutional ethics committee, and all participants were from Guangzhou University of Chinese medicine hospital).

### Primer and probe design

Primer 5.0 software was utilized to design the corresponding primers and detection probes. The *PAH* gene mutations detected include the following sites: R111X (c.331C → T), R176X (c.526C → T), Ex6–96A > G (c.611A → G), and R241C (c.721C → T).), R243Q (c.728G → A), R252Q (c.755G → A), Y356X (c.1068C → A), V399 V (c.1197A → T), R413P (c.1238G → C). In this study, five pairs of primers were designed to amplify the 9 mutation site fragments. A total of 18 probes were designed, of which 9 probes were used to detect the mutation sites and the other 9 probes were normal control probes. The 5′ end of the primer was biotin labeled.

**Fig. 1:**
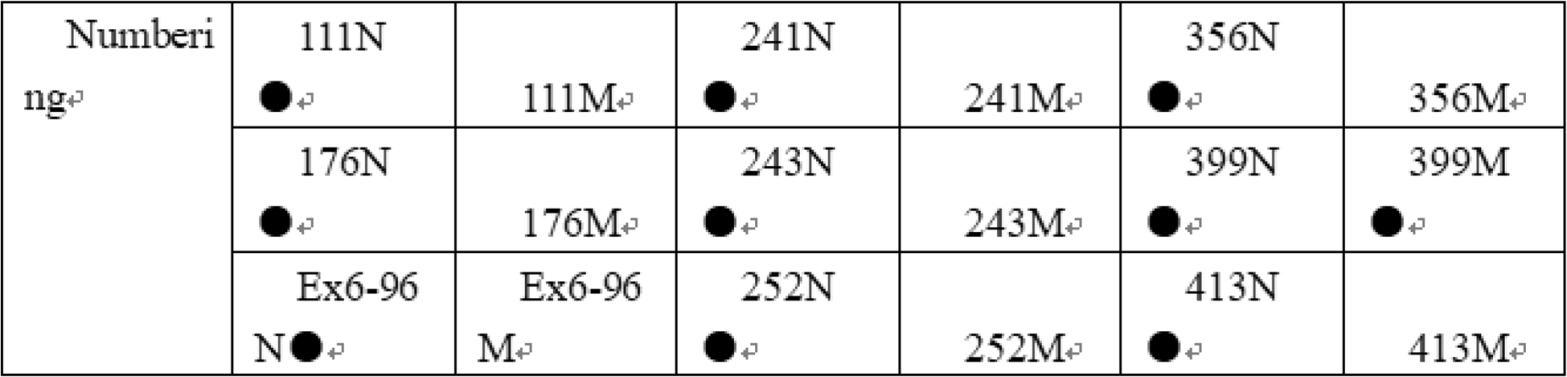
Schematic layout of the membrane probe

The designed *PAH* wild-type gene and mutant detection probes both crossed the point mutation site, and their 5’ends were aminated (5′-NH_2_). The sequence of primers and probes is illustrated in Tables 1 and 2. Primers and probes were synthesized by the Shanghai Bioengineering Company. The layout of the DNA probe on the carboxy nylon membrane is shown in Fig. 1.

**Table 1:** Primer sequences and positions of single-tube multiplex PCR amplification of PAH gene fragments

Primer name	Sequence (5′-3′)	Detection site	Length (bp)
PAHF1	GCTAGCCTGCCTGCTCT	R1 11X	145
PAHR1	GAAGACAGTGTGGAGTTACTT		
PAHF2	TGCCCTGCTTGAGACACC	R176X, Ex6–96A > G	191
PAHR2	CATGGAAGCCACAGTAC TT		
PAHF3	CTAGCGTCAAAGCCTATGT	R241C, R243Q, R252Q	338
PAHR3	ATGAACCCAAACCTCATTC		
PAHF4	TATGGGATGCAGCAGGG	Y356X, V399 V	274
PAHR4	ACCACCCACAGATGAGTGG		
PAHF5	TTCTCCAAATGGTGCCCTT	R413P	232
PAHR5	GCGATGGTAGGGAAAGACAG

**Table 2:** Sequence of site detection probes

Detection site	Normal probe sequence (5′-3′)	Mutation probe sequence (5′-3′)
R111X (C → T)	GAGCTTTCACGAGATAAGAA	ATGAGCTTTCATGAGATAAG
R176X (C → T)	CATCCCTCGAGTGGAA	GCCCATCCCTTGAGTG
Ex6–96 (A → G)	TGTACTCATAGCAAGCAT	ATTGTACTCACAGCAAGC
R241C (C → T)	TGGTTTCCGCCTCCG	GGTTTCTGCCTCCGAC
R243Q (G → A)	CACAGGTCGGAGGCG	ACAGGTTGGAGGCGGA
R252Q (G → A)	GAAATCCCGAGAGGAAAG	AAGAAATCCTGAGAGGAAAG
Y356X (C → A)	CCTACAGTACTGCTTATCA	GCCTACAGTAATGCTTATCA
V399 V (A → T)	CACCTCACCTTACTTTCTC	ACCTCACCTAACTTTCTCC
R413P (G → C)	TCAGTTCGCTACGACC	CTTCTCAGTTCCCTACGA

### Preparation of hybrid membrane strips

In this study, biotinylated PCR products were hybridized with oligonucleotide probes on the solid phase to achieve PAH genotyping.

The molecular hybrid membrane was prepared as follows: The carboxyl nylon membrane (Pall Corporation, USA) was acidified with 0.1 mol/L HCl for 10 min, and the nylon membrane was activated with ethyldimethylaminopropyl diimine (Sigma, USA) for 10 min. Rinsing the membrane with distilled water twice and air dried for 20 min. Then, according to the layout of the probe on Fig. 1, 0.5 μmol/L oligonucleotide probe was diluted with 0.5 mol/L NaHCO_3_ -Na_2_CO_3_ buffer (pH 8.0) at the corresponding position. Dried in the air for 20 min, and then soaked in a nylon membrane with 0.1 mol/L NaOH for 10 min. After rinsing with distilled water, dry at room temperature for 10 min.

### PCR amplification of PAH gene fragments

The PCR conditions were as follows: In a 25 μL PCR reaction system, 1 μL PCR buffer, 400 μmol/L dN(U)TP, and 0.2 μmol/L PAH primers, and 2 U Taq enzyme (Takara, China) and 0.5 U UNG enzyme (Thermo, USA) were used. The PCR reaction was performed on a T100 PCR machine (Bio-rad, USA). The reaction conditions were incubation at 50 °C for 15 min, predenaturation at 95 °C for 10 min, then 95 °C, 30s; 57 °C, 30s; 72 °C, 30s for 40 cycles, and 72 °C for 5 min.

After the amplification was completed, 5 μL of the PCR product and 5 μL of DL2000 marker (Takara, China) were electrophoresed on a 1.5% agarose gel. Observe the electrophoresis result on a gel imager (UVP, USA).

### Molecular hybridization analysis

Molecular hybridization assays include hybridization, washing, incubation, washing, and color development. The hybridization solution used for molecular hybridization analysis is presented in Table 3.

**Table 3:** Hybridization components used in molecular hybridization analysis

Hybrid fluid	Ingredient
Liquid A	2x SSC, 0.1% SDS
Liquid B	0.5 x SSC, 0.1% SDS
Liquid C	0.1 mol L Sodium citrate
Incubating liquid	0.25 U/mL Streptomycin –POD, 2x SSC, 0.1% SDS
Color-substrate solution	0.0015% hydrogen peroxide, 0.1 mg/mL TMB, 0.1 mol/L Sodium citrate

Note: Streptavidin-POD was purchased from Thermo, USA

Hybridization: a membrane strip was placed in a hybridization tube containing 10 mL of Liquid A. Single-tube multiplex PCR amplified products were added. After treatment at 100 °C for 10 min, hybridization was performed in a molecular hybridization oven (Yonon YN-H16, China) at 45 °C for 2 h. At the same time, added about 45 mL of Liquid B to a 50 mL centrifuge tube and placed in a hybridization chamber for preheating.

Washing the membrane: After the hybridization is completed, the membrane strip was moved to the liquid B and the membrane was washed at 45 °C for 10 min.

Incubation: Prepare the incubating liquid and incubate the membrane strip in it for 30 min at room temperature.

Washing the membrane: Discard the incubating liquid, add Liquid A and gently wash the membrane twice at room temperature for 5 min each time. After draining, add Liquid C and wash the membrane for 2 min at room temperature.

Color development: Prepare a Color-substrate solution, soaked the membrane strip in it and protected from light for 10 min. After the color development is completed, wash the membrane with deionized water once to observe the result.

It is judged by whether a blue spot signal appears at the test site or not. It’s indicating that the sample is wild type when all normal sites (N) on the membrane were developed and all mutation sites (M) were not colored. On the contrary, Mutation sites (M) appeared on the strip indicates that the site is positive.

### Repeatability and verification test

Ten DNA samples of the 10 (including wild-type) PAH genotypes mentioned above were taken and replicated within the batch (3 times) and inter-assay (10 days) tests to determine the repeatability of the PCR-Reverse Dot Blot method. One hundred eighty PKU-deficient and 20 PKU-normal DNA samples were tested by DNA sequencing and the established method in the double-blind experiment to assess specificity and accuracy.

## Results

### PCR amplification result of *PAH* gene fragment

**Fig. 2:**
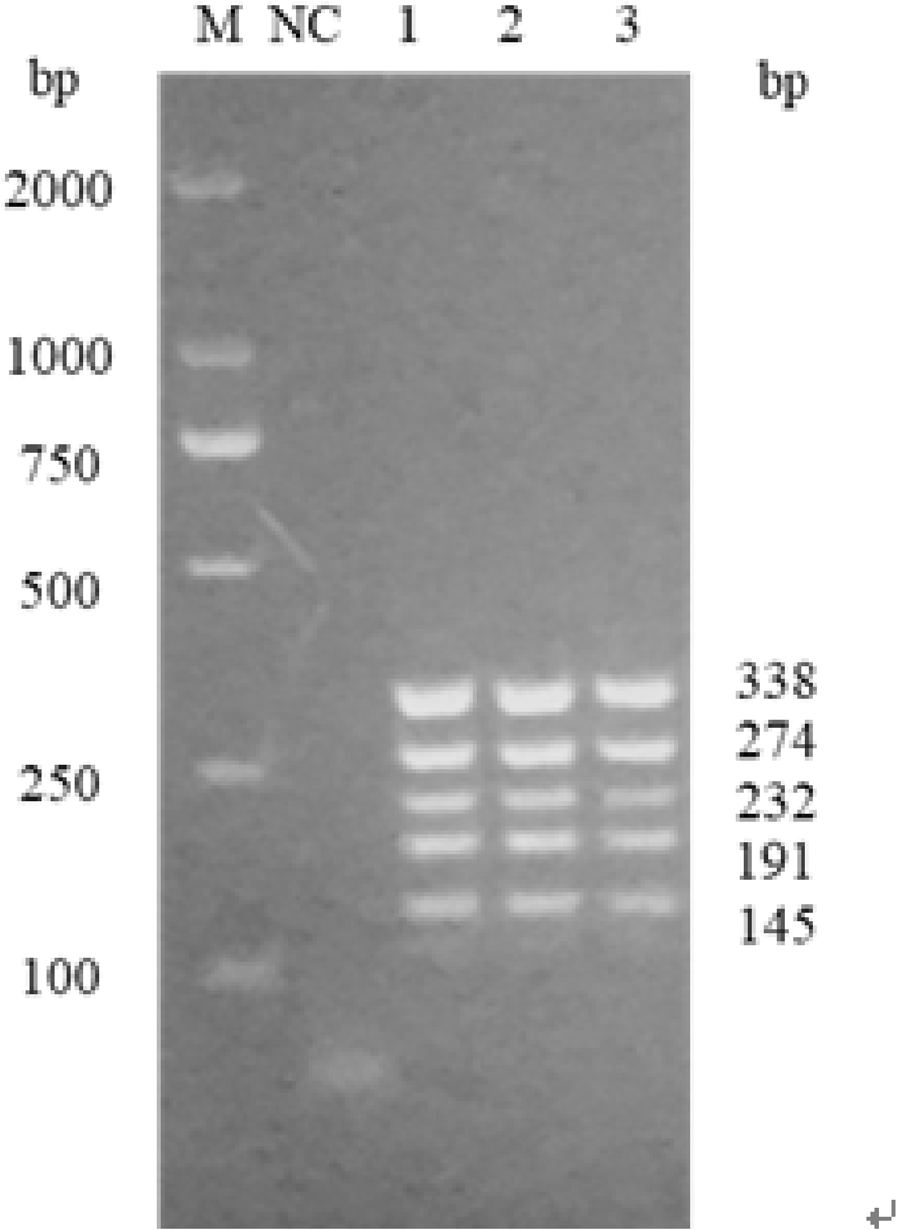
The result of single tube multiplex PCR amplification of the PAH gene fragment

Using multiplex PCR in a single tube, we amplified PAH gene fragments of the detection loci whose size is 338 bp, 274 bp, 232 bp, 191 bp, and 145 bp respectively. The electrophoresis band is clear and without smear, indicating that the established PCR system can efficiently amplify PAH gene fragments of blood DNA (Fig. 2).

### The result of common PAH gene mutation loci in the Chinese population detected by reverse dot blot

**Fig. 3:**
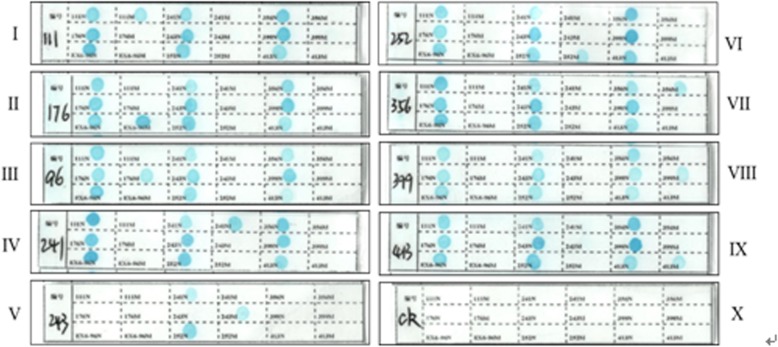
Representative results detected by reverse dot hybridization of 9 common PAH gene mutations in the Chinese population

The representative results of 9 common mutations in the PAH gene in the Chinese population (Fig. 3) are detected by the established method that a single-tube multiplex PCR amplification combines with reverse dot blot. After the hybridization and coloration are completed, check whether the wild-type control spot on the dot blot membrane shows a clear blue round spot. For a sample to be tested, if all the wild-type control spots are colored while no blue spots appear in the mutant control spot, it can be determined that none of the above 9 PAH gene mutations have been detected. Similarly, it is suggested that the mutant allele is heterozygous if all wild-type control spots and some specific mutation site on the hybridized membrane strip present a clear blue spot. Meanwhile, it is suggested that the test fails when wild-type control spots and mutational spots are not colored simultaneously, then it needs to be retested after the analysis of the cause.

The 10 known PAH genotypes (including wild-type) using the method above were validated by DNA sequencing analysis. The results of PCR-Reverse Dot Blot are consistent with DNA sequencing analysis results, it’s shown that the detection method, PCR amplification combined with reverse dot hybridization technique, established in this study can be used to detect common mutants of PAH.

### Evaluation of methodology

The repetitive test results showed that hybridized spots have a consistent color depth detected in wild-type and mutation PAH samples by the above method.

As for the result of the inter-batch repeat experiment, due to different room temperature, it has a certain degree of color depth difference but it does not affect the interpretation of results. Before methodological comparison, the designed probes and primers should be validated in multiple samples to ensure that the color of each point is uniform and visible to the naked eye.

The specificity and accuracy of the PCR-RDB technique were evaluated using 200 DNA samples of known PAH genotypes. A double-blind controlled trial was conducted so that neither the researchers nor the participants knew who they had taken. And the results of the double-blind controlled trial show that the genotypes of all DNA samples detected by this technique were identical to the results of the DNA sequencing analysis, with 100% specificity and accuracy.

In this study, the frequencies of different genotypes were shown in Table 4. Frequencies of R111X, R176X, Ex6–96A, R241C, R243Q and R252Q genotypes were 8, 0.5, 16.5, 1.5, 27, 4.5%. All the samples tested in this study belong to a single mutation.

**Table 4:** The frequencies of different genotypes of PAH

Genotypes	The number of cases(%)
R111X	16 (8%)
R176X	1 (0.5%)
Ex6–96A	33 (16.5%)
R241C	3 (1.5%)
R243Q	54 (27. %)
R252Q	9 (4.5%)
Y356X	26 (13%)
V399 V	21 (10.5%)
R413P	17 (8.5%)
Wild-type	20 (10%)
Total	200

**Fig. 4:**
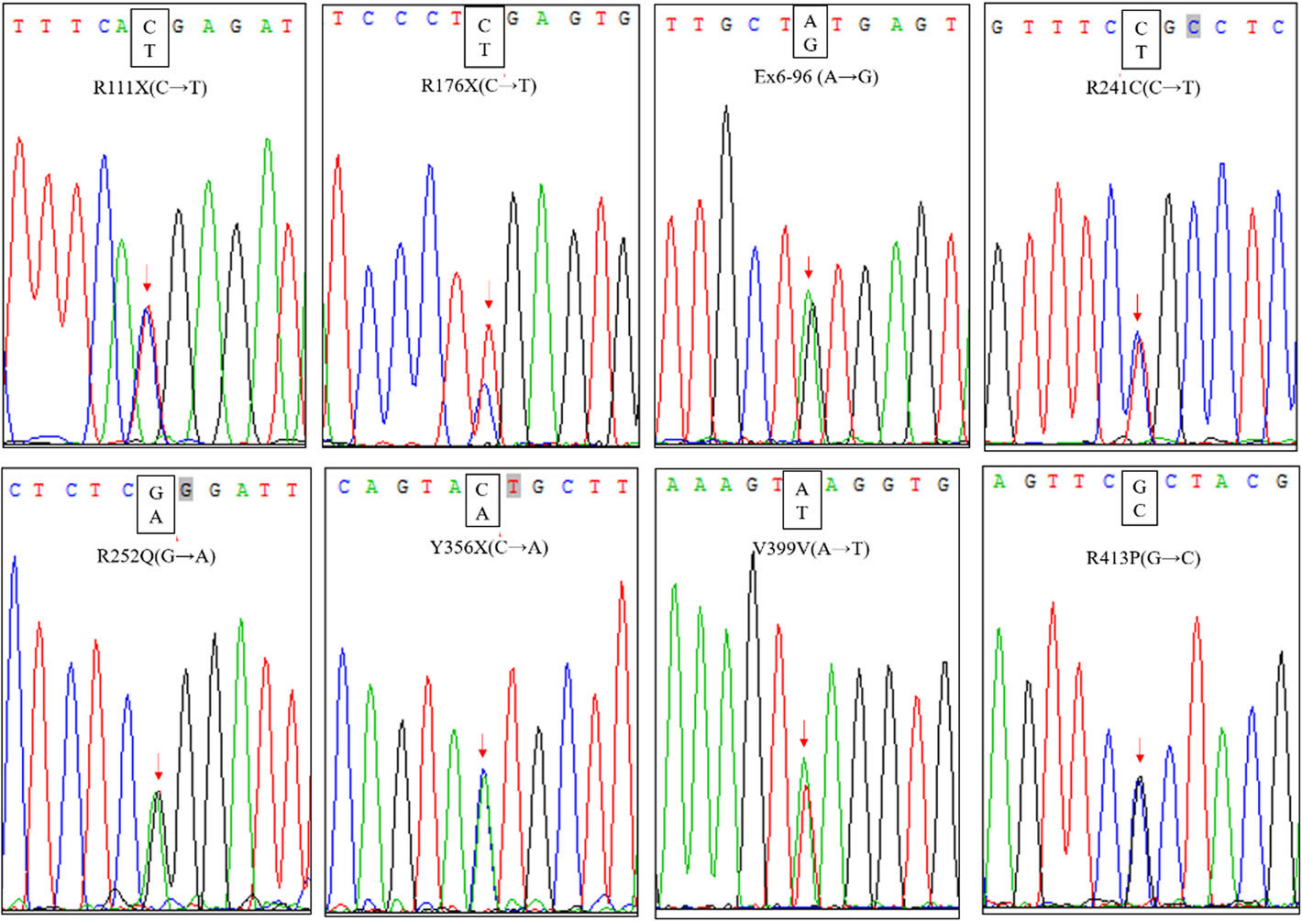
Representative results of DNA sequencing analysis of PAH gene mutation

DNA sequencing results of partial samples were shown in Fig. 4.

## Discussion

Phenylketonuria is an amino acid metabolic disease that is mainly caused by a decrease or absence of PAH activity in the human body. The function of PAH requires the use of tetrahydrobiopterin (BH_4_), so PKU is divided into typical PKU and BH_4_ deficient PKU, the former accounted for 98 to 99% [9]. Typical PKU patients mainly use the low-phenylalanine diet, while BK_4_ PKU patients use BH_4_ therapy [10, 11]. After the clinical screening of neonates with fluorescence quantitative methods, an accurate, rapid and sensitive gene detection method is still needed to establish to provide evidence for the clinical diagnosis.

At present, PCR-restriction fragment length polymorphism (PCR-RFLP), PCR-single strand conformation polymorphism(PCR-SSCP), denaturing high-performance liquid chromatography (DHPLC), direct sequencing and other techniques are mainly applied to detect PAH gene mutations in China [12–14]. The traditional PCR method system lacks the dUTP&UNG enzyme against contamination, and the amplification product needs to be digested by the restriction endonuclease, sequenced, etc. The operation is cumbersome, which increases the risk of pollution, and lacks intuitiveness. Single-tube multiplex PCR combined with RDB technology is based on membrane-bound allele-specific oligonucleotide probes for molecular hybridization with genomic DNA-specific amplified fragments. Point mutations or small deletions or insertions within a gene are detected directly by a chemical chromogenic reaction. Compared with other PAH gene mutation molecular diagnostic methods, the method established in this study has the following advantages: The method requires simple techniques and experimental conditions, low cost, operability, and economy. Moreover, RDB technology is more mature and has been widely used in the molecular diagnosis of genetic diseases such as thalassemia and G6PD in China^7^. This method has great developmental advantages in detecting the number of mutations. With the discovery of new mutations in the PAH gene, corresponding oligonucleotide probes can be easily added to the membrane to increase the detection throughput. Therefore, this method is suitable for clinical application and screening of PAH genes in large populations.

## Conclusions

In this study, the RDB technique was used to detect 9 common mutation- sites in the *PAH* gene in the Chinese population. The experimental results are in full compliance with the sequencing results. This method is characterized by good repeatability, high specificity, and high accuracy. It can detect 9 common PAH gene mutations in the Chinese population in one PCR reaction and one molecular hybridization within one working day. The method is cheap and convenient. This research provides a simple, rapid and accurate gene detection method for clinical screening of large populations of PAH gene mutations.

## Data Availability

All data generated or analyzed during this study are included in this published article and its supplementary information files.

## Abbreviations

BH_4_: Retrahydrobiopterin
DNA: Deoxyribonucleic acid
G6PD: Glucose-6-phosphate dehydrogenase deficiency
HCl: Hydrochloric acid
Na_2_CO_3_: Sodium Carbonate
NaHCO_3_: Sodium Bicarbonate
NaOH: Sodium hydroxide
PAH: Phenylalanine hydroxylase
PCR: Polymerase Chain Reaction
PKU: Phenylketonuria
R111X, R176X, Ex6–96A > G, R241C, R243Q, R252Q, Y356X, V399 V, R413P: Represent different PAH mutations
RDB: Reverse dot blot
